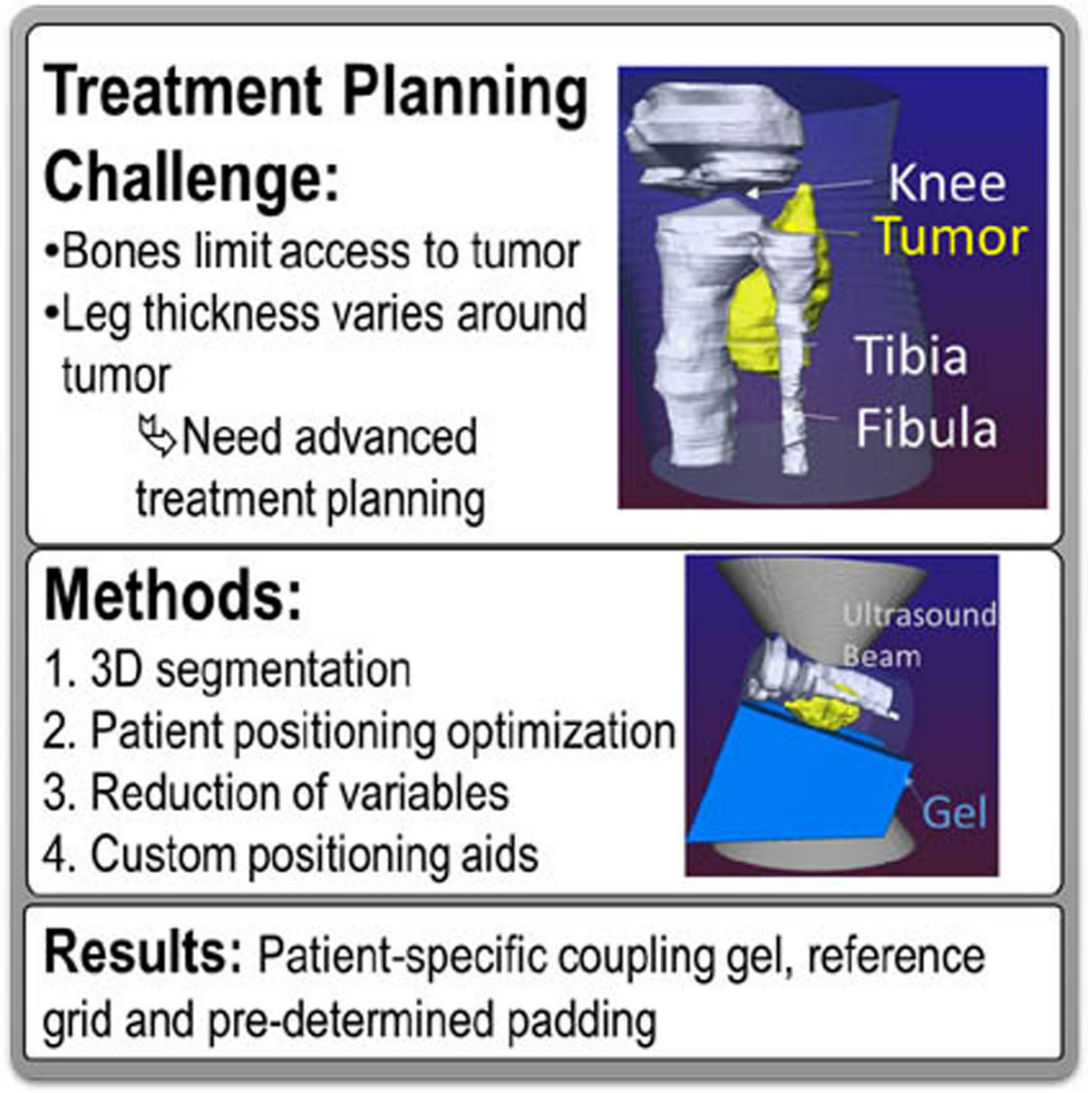# Treatment planning and patient positioning for MR-guided high intensity focused ultrasound treatment: a systematic approach

**DOI:** 10.1186/2050-5736-3-S1-P65

**Published:** 2015-06-30

**Authors:** David Kinnaird, Doug Wackerle, Daniel Yang, Matthew Oetgen, Avinash Eranki, AeRang Kim, Karun Sharma, Harry Kim, Peter Kim, Pavel Yarmolenko, Haydar Celik

**Affiliations:** 1 Children’s National Health System, Washington, D.C., United States; 2 The George Washington University School of Medicine, Washington, D.C., United States; 3 Princeton University, Princeton, New Jersey, United States; 4 Texas Scottish Rite Hospital for Children, Dallas, Texas, United States

## Background/introduction

Treatment duration as well as time spent on patient positioning imposes limitations on therapeutic use of MR-guided High Intensity Focused Ultrasound (MR-HIFU). Reduction of overall treatment time is especially important in potential pediatric applications and in other cases where general anesthesia must be used, due to the risks associated with prolonged anesthesia. Typically, up to 4 hours are allotted for the procedure, with patient positioning and treatment planning requiring an hour or more. If re-positioning is required during treatment, acquisition of needed images and re-planning of treatment may require 30 minutes or longer before ablation can resume. These delays limit the total time allowed for treatment, limiting the size of tumors that can be treated and increasing the risks as well as the cost of the procedure. The aim of this study is to evaluate the information needed to accurately plan MR-HIFU ablation of solid extremity tumors and to rationally design a practical approach to patient positioning for such treatments.

## Methods

Correct positioning of a tumor-bearing limb was accomplished via three methods that rely on three-dimensional segmentation of pre-procedural MR images: 1) a printed, concise, patient-specific guide that shows all steps necessary for optimal patient positioning, 2) a printed or projected grid that is spatially referenced to the printed guide, and 3) a patient-specific ultrasound stand-off gel pad that accurately orients the targeted extremity relative to the center of the MR-HIFU tabletop membrane. Especially complex cases from a set of 41 patients were examined in development of these methods. Performance characteristics of one of the MR-HIFU devices currently in clinical trials, the Philips Sonalleve V2 (Philips Healthcare, Vantaa, Finland) were used.

## Results and conclusions

The methods proposed in this study present practical and time saving approaches to MR-HIFU treatment planning. Patient specific stand-off gel allows the determination of the angle and distance of the target extremity to be made prior to treatment. The patient-specific measurements can be used to set the height and angle of the target limb relative to the HIFU tabletop membrane, allowing for pre-treatment preparation of an ultrasound stand-off gel pad that optimally positions the limb for treatment. The coordinate algorithm and the grid further lock in the limb position and may allow for more thorough treatment pre-planning in future studies. These methods take a systematic approach to reducing total treatment time for MR-HIFU ablations and they may allow for treatment of a wider variety of lesions. Analysis of especially challenging solid tumors of the extremity from our institution demonstrate that the approach is promising and warrants further study.

**Figure 1:**